# Certolizumab-induced sarcoidosis in a patient with psoriatic arthritis – a case report and review of literature

**DOI:** 10.1007/s00296-024-05680-8

**Published:** 2024-08-24

**Authors:** Małgorzata Biernikowicz, Weronika Pilch, Wiktoria Wojturska, Mariusz Korkosz, Jarosław Nowakowski

**Affiliations:** 1 Students’ Scientific Group of Rheumatology and Immunology, Jagiellonian University Medical College, Krakow, 30-688 Poland; 2 Department of Rheumatology and Immunology, Jagiellonian University Medical College, ul. Jakubowskiego 2, Krakow, 30-688 Poland

**Keywords:** Drug-induced sarcoidosis-like reaction, Sarcoidosis, Certolizumab, Tumour necrosis factor-α antagonists

## Abstract

Tumour necrosis factor-α (TNF- α) antagonists are considered a significant therapeutic option in the treatment of sarcoidosis. Nevertheless, their use can also paradoxically result in sarcoidosis-like reactions. Here, we present a case of a 56-year-old patient with psoriatic arthritis who after 3 months of certolizumab therapy developed pulmonary sarcoidosis. Therefore, certolizumab was discontinued and prednisone initiated. Subsequently, 4 months later a complete remission of interstitial lesions was observed. Due to insufficient control of psoriatic arthritis, upadacitinib and methotrexate were prescribed and despite initial improvement, a couple of months later a massive exacerbation of skin psoriasis occurred and the treatment was switched to secukinumab. As of today, no evidence of sarcoidosis recurrence has been noted. Drug-induced sarcoidosis-like reactions (DISR) appear to be less frequently associated with certolizumab rather than with other anti-TNF-α agents. However, specific mechanisms of this phenomenon remain unclear and require future investigation.

## Introduction

Sarcoidosis is a multisystem disorder of unknown cause characterised by the presence of noncaseating granulomas. Lung involvement is the most common manifestation, occurring in more than 90% of patients, with approximately 43% presenting respiratory symptoms [1, 2]. Other frequently affected organs include skin, eyes, joints, liver, and spleen [2–4]. In Europe, the mean age at diagnosis is around 40 years, with a male-to-female ratio varying between regions, from 1:1 in Northern to 1:2 in Southern Europe [5]. To accurately identify sarcoidosis, it is crucial to consider differential diagnoses such as tuberculosis, fungal infections and chronic beryllium disease [6]. Furthermore, literature reports have associated sarcoidosis with the use of medications such as immune checkpoint inhibitors, highly active antiretroviral therapy, interferons, and tumour necrosis factor-alpha (TNF-α) antagonists [7]. While few case reports of sarcoidosis following certolizumab treatment exist, this article presents a case of certolizumab-induced sarcoidosis in a male with psoriatic arthritis and reviews the available literature on the subject.

## Case presentation

In October 2022, a 56-year-old male presented at the outpatient rheumatological department of University Hospital in Cracow for an emergency visit. Earlier that month, he was admitted to a local hospital. Initially, the patient exhibited signs of asthma exacerbation, including acute dyspnea and a dry cough, but he was unresponsive to bronchodilators with no apparent bronchospasm. Chest X-ray revealed hilar lymphadenopathy. Due to respiratory insufficiency, the patient required oxygen therapy; however, glucocorticoids (prednisone 40 mg daily, tapered over the next months) alleviated his symptoms. The QuantiFERON-TB Gold test and bacteriological and mycological evaluation of bronchoalveolar lavage were negative. Fungal infections presenting with granulomas, such as coccidioidomycosis and histoplasmosis, were considered highly unlikely in Poland as the patient had no history of foreign travel, and thus were excluded from the differential diagnosis. There was no evidence of foreign bodies or beryllium exposure in the patient’s history. Pulmonary function tests showed an FEV1 of 77% of the normal value and an FEV1/FVC ratio of 63.8%. A subsequent chest CT scan revealed bilateral hilar changes, areas of ground-glass opacity, interlobular septal thickening, and bronchial wall thickening. Histopathological examination of samples obtained from endobronchial ultrasound-guided transbronchial needle aspiration (EBUS-TBNA) showed diffused lymphocytes, groups of epithelioid histiocytes, and non-caseating epithelioid-cell granulomas. The Ziehl-Neelsen stain was negative. There were no other clinical symptoms or laboratory abnormalities suggesting involvement of other organs. Abdominal ultrasound, ECG and ophthalmologic examination were normal. Therefore, a diagnosis of pulmonary sarcoidosis was established. In the outpatient rheumatological clinic, an association between sarcoidosis and certolizumab treatment was recognised. The patient had comorbidities including psoriasis, well-controlled asthma without medication, hypercholesterolemia, and prediabetes. He had been treated for psoriasis since 1991 with various medications, including acitretin, cyclosporine, PUVA, methotrexate, methylprednisolone, adalimumab, ixekizumab, and ustekinumab. In March 2022, he was diagnosed with psoriatic arthritis involving axial skeleton and peripheral joints. He was treated with methotrexate (reinitiated) and etoricoxib. Due to unsatisfactory response (BASDAI 6,1; VAS 7/10; PASI 18; BSA 25,5%), certolizumab treatment was initiated in July 2022 (without methotrexate), resulting in reduced disease activity. However, three months later, the patient developed pulmonary sarcoidosis necessitating therapy with prednisone (40 mg daily) which was associated with use of certolizumab, eventually discontinued. Two months later, a follow-up chest X-ray showed regression of hilar lymphadenopathy, and methotrexate treatment (25 mg s.c. once a week) was reinstated. By February 2023, after 4,5 months of steroid treatment, complete remission of interstitial changes in control CT scan was achieved, and prednisone was withdrawn. Due to poor psoriatic arthritis control, upadacitinib therapy was initiated, leading to arthritis remission but persistence of skin lesions. After 8 months, the patient experienced a severe psoriasis flare-up, requiring hospitalization and therapy switch to secukinumab which allowed for sustaining low disease activity.

## Discussion

The aetiology of sarcoidosis is complex and not fully understood. Major factors that may contribute to the development of this disease include genetic susceptibility [8], various environmental exposures [9], infectious agents, particularly mycobacteria [10], and autoimmunity [11]. Moreover, not all patients with sarcoidosis require systemic therapy; it should be reserved for those at increased risk of death, permanent disability or to improve quality of life [12, 13]. Glucocorticoids are considered as a first-line treatment for severe pulmonary sarcoidosis. In cases of significant glucocorticoid-related adverse events, insufficient response, or relapse, other immunosuppressive drugs such as methotrexate, azathioprine, or leflunomide can be added [13]. For refractory sarcoidosis, TNF-α antagonists are regarded as an effective third-line therapy option [14].

Sarcoidosis as well as psoriatic arthritis are inflammatory diseases characterised by an involvement of Th17 cells in their aetiopathogenesis [15, 16]. As of now, there are only a few reports in the literature about the co-occurrence of those two disorders. A study conducted by Mazzucchelli et al. aimed to find the possible association between spondylarthritis and sarcoidosis. They discovered that the incidence of sarcoidosis in people with spondylarthritis was higher than that in the control group (adjusted OR = 1.50 (95% CI: 1.14–1.97), specifically in patients with psoriatic arthritis, adjusted OR = 1.81 (95% CI: 1.29–2.55)) [17]. Although in the majority of cases psoriatic arthritis precedes the diagnosis of sarcoidosis, we found a case report of a 78-year-old woman, in whom the sequence of those two entities was reversed. She had family history of psoriasis, was diagnosed with sarcoidosis at 60 years old and over a decade later developed symptoms of psoriasis and subsequently psoriatic arthritis [18].

Care report was described in line with the CARE standard: https://www.carestatement.org/checklist. Searches through Medline/PubMed, Embase, Web of Science, and Directory of Open Access Journals (DOAJ) were conducted on June 3, 2024, without time restrictions. Databases were searched using the following keywords: “certolizumab-induced sarcoidosis”, “certolizumab” AND (“sarcoidosis” OR “sarcoidosis-like” OR “drug-induced sarcoidosis-like reaction” OR “DISR” OR “sarcoidosis reaction”)^9^. Up to the best of our knowledge, there are eight reports of certolizumab-induced sarcoidosis. We summarised them in Table 1. However, it is important to emphasise that the number of such cases in the literature may be underestimated. Aubart et al. searched the World Health Organization pharmacovigilance database (VigiBase) and identified 25 reports of certolizumab-induced sarcoidosis between the years of 1967 and 2019, but we were unable to access the specific clinical characteristics of these patients [19]. Moreover, in relation to other anti-TNF-α agents such as infliximab, etanercept and adalimumab, sarcoidosis after certolizumab seems to be less-commonly reported [19–21].

**Table 1:** Reported cases of certolizumab-induced sarcoidosis

Article	Age	Sex	Sarcoidosis manifestation	Time to the occurrence of sarcoidosis symptoms	Medications used in the treatment of sarcoidosis	Follow-up	Certolizumab treatment indication
Donzella et al. 2024 [22]	40	F	Uveitis, pulmonary, mediastinal, and hilar, lymphadenopathy, arthralgia	10 months	Topical steroids	Two months, complete resolution of pulmonary findings	Psoriasis
Donzella et al. 2024 [22]	46	F	Pulmonary, mediastinal lymphadenopathy, arthralgia	3 months	Prednisone 30 mg daily	No data	Spondylarthritis
Hum et al. 2022 [23]	55	M	Renal	7 months	Prednisolone 80 mg daily	Twenty one months, still on 5 mg of prednisolone daily, in remission of sarcoidosis	Psoriatic Arthritis
Koda et al. 2020 [20]	69	F	Systemic - both lungs and bilateral hilar and mediastinal lymphadenopathy, uveitis, subcutaneous nodule on right knee	6 years	Prednisolone 20 mg daily	Six months, still on 10 mg of prednisolone daily without recurrence of sarcoidosis	Rheumatoid arthritis
Moisseiev and Shulman, 2014 [24]	64	F	Ocular - uveitis	3 years	Topical steroid drops, anti-glaucoma medication, periocular injections of 40 mg triamcinolone acetonide (persistent macular oedema)	Two months, the uveitis had decreased and visual acuity had improved. One year, no recurrence of uveitis, partial improvement of macular oedema.	Rheumatoid arthritis
Sakai et al. 2017 [25]	63	M	Cutaneous	3 months	Topical glucocorticoid therapy	No recurrence	Rheumatoid arthritis
Toussirot et al. 2019 [26]	45	F	Renal, pulmonary, mediastinal, supraclavicular, hilar and retroperitoneal lymphadenopathies, spleen	6 months	Prednisone 60 mg daily, (methotrexate 15 mg weekly one month after the occurrence of retroperitoneal lymphadenopathy and splenic involvement)	Fifteen months - improvement of renal function and resolution thoracic inflammatory lesions, 8 months after the occurrence of retroperitoneal lymphadenopathy and splenic involvement total normalisation of PET CT was observed	Non-radiographic axial spondylarthritis
Yilmaz et al. 2024 [27]	56	F	Bilateral cervical, hilar lymphadenopathy, arthralgia	After 5 doses	No data	No data	Ankylosing spondylitis

Only one of the cases mentioned in Table 1 as well as our patient’s, were associated with certolizumab-induced sarcoidosis after treating psoriatic arthritis. Hum et al. described history of a 55-year-old male with renal manifestation of sarcoidosis after 7 months of therapy with certolizumab. Similarly to our patient, he developed psoriasis in his twenties and afterwards psoriatic arthritis. In both cases, the resolution of sarcoidosis symptoms was observed after the discontinuation of certolizumab and treatment with glucocorticoids. Whereas, considering all sarcoidosis induced by TNF-α antagonists in treatment of psoriatic arthritis, the number of patients included in the review conducted in 2021 by Rodrigues-Fernandes et al. was 15 [21].

Anti-TNF-α agents play a significant part in therapy of various inflammatory disorders such as rheumatoid arthritis, ankylosing spondylitis, psoriatic arthritis, plaque psoriasis, juvenile idiopathic arthritis, ulcerative colitis, Crohn’s disease, and sarcoidosis [14, 28–33]. However, they can also be associated with the development and exacerbation of these diseases, in a so-called paradoxical reaction. The most common manifestations of such effect include psoriasis and psoriasiform lesions, uveitis, inflammatory bowel diseases, vasculitis, systemic lupus erythematosus, interstitial lung disease as well as sarcoidosis (also known as drug-induced sarcoidosis-like reaction (DISR) [34, 35]. DISR can be defined as a systemic granulomatous syndrome that occurs in a temporal relation after treatment initiation with specific drugs. Four most frequent groups of medications associated with DISR are immune checkpoint inhibitors, highly active antiretroviral therapy, interferons, and TNF-α antagonists [7].

Rodrigues-Fernandes et al. in their review identified 107 cases of DISR after TNF-α antagonists, with most of them related to the use of etanercept (48,6% of patients) [10]. The difference in the incidence of DISR after treatment with various anti-TNF-α agents can be attributed to a number of diverse pathomechanisms. Although not fully understood, some possible ones include cytokine imbalance due to long term TNF-α suppression, excess IFN-α and IFN-γ expression in dendritic cells resulting in a shift towards a Th1/Th2 profile, or a preferential induction of Th17 over Treg cells causing an elevated production of autoantigens which in turn leads to a paradoxical reaction [36–38]. A possible link between an underlying infectious agent and DISR was also proposed, especially as anti-TNF-α therapy predisposes to infections [39].

Certolizumab is a TNF-α-neutralising humanised antigen-binding fragment of a monoclonal antibody that does not consist of an Fc region and as such it cannot result in a cytotoxic complement-induced cell lysis. In a series of in vitro studies Nesbitt et al. aimed to examine mechanisms of action of various.

TNF-α antagonists. They concluded that in contrast to infliximab, adalimumab and etanercept, certolizumab did not induce myeloperoxidase release as well as apoptosis in peripheral blood mononuclear cells and in human polymorphonuclear neutrophils. Furthermore, certolizumab unlike etanercept, inhibited the production of lipopolysaccharide-induced IL-1β [40]. Additionally, the increased expression of IL-1β can be observed inside granulomas in patients with sarcoidosis [41]. Moreover, antibodies produced against certolizumab may cause inadequate TNF-α suppression [20]. Vermeire et al. summarised rates of anti-drug antibodies (ADA) formation to different anti-TNF-α agents and found that the highest rates of ADA were associated with either infliximab or adalimumab (respectively 0.0–65.3% and 0.3–38.0%), while the percentage of patients with ADA to certolizumab was lower (3.3–25.3%) [42]. In another study certolizumab serum concentration highly correlated with the drug effectiveness in TNF-α neutralisation but no connection with serum level of ADA against certolizumab was noted [43]. The aforementioned mechanisms may play a role in the development of certolizumab-induced DISR.

## Conclusion

In conclusion, the case presented underscores the importance of vigilance regarding potential adverse effects of TNF-α antagonist therapy including also certolizumab, in patients with inflammatory conditions such as psoriatic arthritis. While these medications play a crucial role in managing various inflammatory disorders, their use can also lead to paradoxical reactions, encompassing the development or exacerbation of sarcoidosis. The association between certolizumab and sarcoidosis, though relatively rare, warrants attention from clinicians. This case serves as a reminder to consider sarcoidosis in the differential diagnosis of patients presenting with respiratory symptoms or radiographic findings suggestive of granulomatous disease undergoing TNF-α antagonist therapy. Further research is needed to elucidate the underlying mechanisms responsible for certolizumab-induced sarcoidosis and to identify potential risk factors that may predispose certain individuals to this adverse reaction. Increased awareness among healthcare providers, coupled with comprehensive monitoring and prompt recognition of symptoms, is essential for the timely management and mitigation of such complications.
